# Expanding the application of a UV-visible reporter for transient gene expression and stable transformation in plants

**DOI:** 10.1038/s41438-021-00663-3

**Published:** 2021-11-01

**Authors:** Guoliang Yuan, Haiwei Lu, Dan Tang, Md Mahmudul Hassan, Yi Li, Jin-Gui Chen, Gerald A. Tuskan, Xiaohan Yang

**Affiliations:** 1 Biosciences Division, Oak Ridge National Laboratory, Oak Ridge, TN 37831 USA; 2 The Center for Bioenergy Innovation, Oak Ridge National Laboratory, Oak Ridge, TN 37831 USA; 3 Department of Plant Science and Landscape Architecture, University of Connecticut, Storrs, CT 06269 USA; 4 National Center for Citrus Improvement, College of Horticulture, Hunan Agricultural University, Changsha, 410128 Hunan China; 5 Department of Genetics and Plant Breeding, Patuakhali Science and Technology University, Dumki, Patuakhali 8602 Bangladesh

**Keywords:** Genetic engineering, Molecular engineering in plants, Genetic vectors, Reporter genes, High-throughput screening

## Abstract

Green fluorescent protein (GFP) has been widely used for monitoring gene expression and protein localization in diverse organisms. However, highly sensitive imaging equipment, like fluorescence microscope, is usually required for the visualization of GFP, limitings its application to fixed locations in samples. A reporter that can be visualized in real-time regardless the shape, size and location of the target samples will increase the flexibility and efficiency of research work. Here, we report the application of a GFP-like protein, called eYGFPuv, in both transient expression and stable transformation, in two herbaceous plant species (*Arabidopsis* and tobacco) and two woody plant species (poplar and citrus). We observed bright fluorescence under UV light in all of the four plant species without any effects on plant growth or development. eYGFPuv was shown to be effective for imaging transient expression in leaf and root tissues. With a focus on in vitro transformation, we demonstrated that the transgenic events expressing 1x *eYGFPuv* could be easily identified visually during the callus stage and the shoot stage, enabling early and efficient selection of transformants. Furthermore, whole-plant level visualization of eYGFPuv revealed its ubiquitous stability in transgenic plants. In addition, our transformation experiments showed that eYGFPuv can also be used to select transgenic plants without antibiotics. This work demonstrates the feasibility of utilizing 1x *eYGFPuv* in studies of gene expression and plant transformation in diverse plants.

## Introduction

Reporter genes and systems have been playing essential roles in biological sciences. Various reporter genes have been developed and adapted to a diverse set of organisms, e.g., *lacZ* in microbes^1^, β-glucuronidase (GUS) in plants^2^, luciferase (LUC)^3^ and green fluorescent protein (GFP)^4^ in both prokaryotic and eukaryotic organisms, to name a few. The application of one or another reporter is mainly dependent on the organism of interest and the availability of the imaging and detection technologies. In plants, GUS, LUC, and GFP are extensively used as the reporters or selectable markers. Technically, GUS, with the presence of glucuronides, and LUC, with the presence of luciferin, can be visualized by naked eyes or under light microscopy (e.g., a luminometer or modified optical microscope), respectively, requiring specific substrates. In contrast, GFP simply requires a fluorescence microscope with no need for specific substrates. Recently, a new reporter, *RUBY*, was developed without the need of using special equipment or substrates^5^. *RUBY*, generating the red pigment betalain, is directly visible by naked eyes. However, the red pigment in the leaf or other tissue is irreversible once it is produced, which may inevitably interfere with biological processes, such as photosynthesis.

In contrast, GFPuv (a GFP variant) was optimized for maximal fluorescence to be observed by naked eyes under UV light instead of using fluorescence microscope. Although multiple GFPuv-related studies have been reported in plants, its applications have been limited. GFPuv has been used for the quantification of the fluorescence of GFPuv-labeled plant pathogens and the indication of gene expression in model species tobacco^6–9^.

**Fig. 1:**
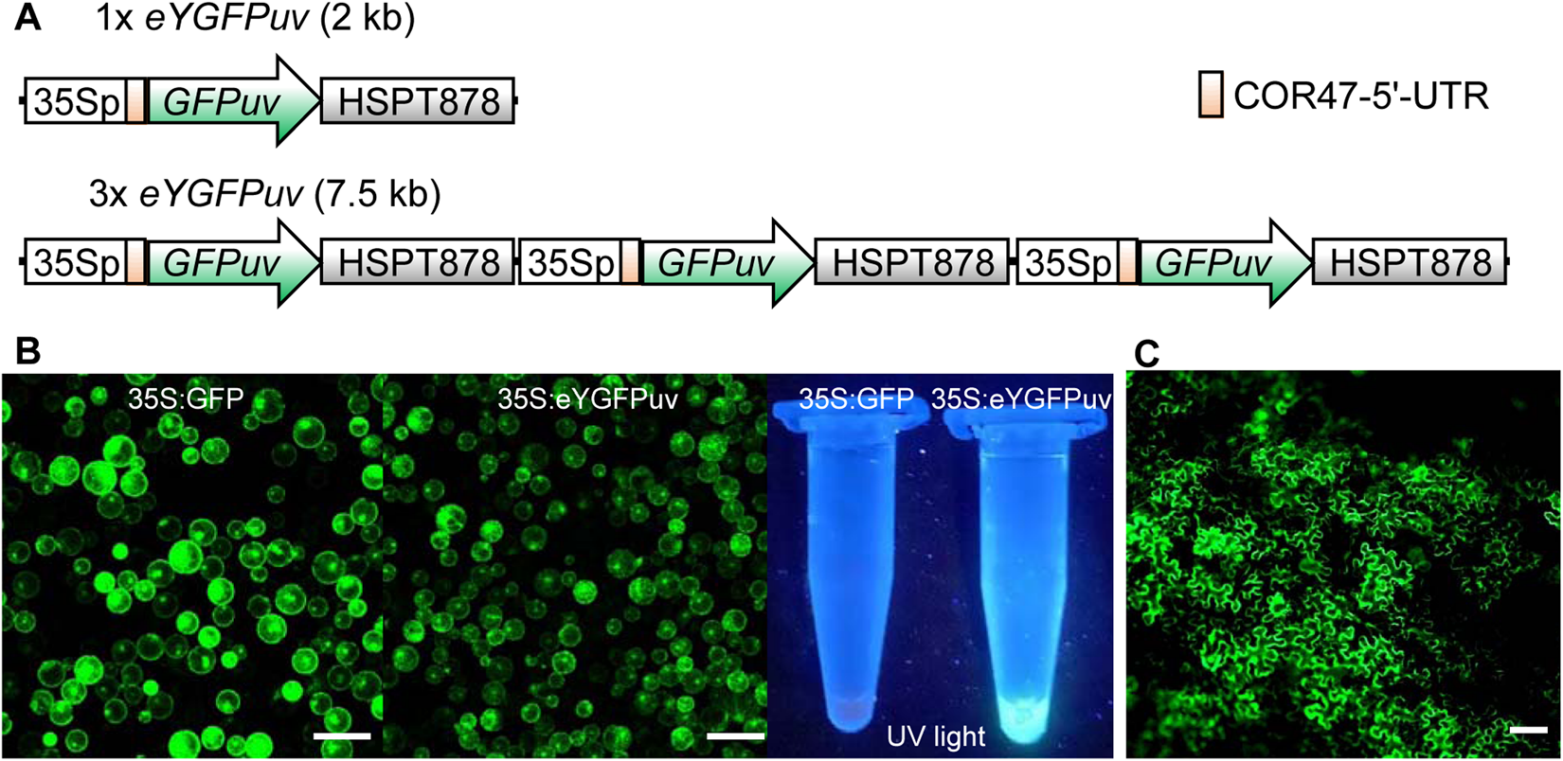
Transient expression of eYGFPuv in the *Arabidopsis* protoplasts and the tobacco leaf. **A** Illustration of 1x *eYGFPuv* and 3x *eYGFPuv*. **B** Visualization of GFP-expression of 35S:GFP and 35S:eYGFPuv in *Arabidopsis* protoplasts under fluorescence microscope and UV light, respectively (Scale bar, 100 μm). **C** Visualization of eYGFPuv expression of 35S:eYGFPuv under fluorescence microscope, three-day post tobacco (*N. benthamiana*) leaf infiltration (Scale bar, 50 μm)

*Agrobacterium*-mediated *in planta* transformation and tissue-culture-based transformation are the most commonly used methods for plant genetic engineering^10–13^. However, one unavoidable issue related to tissue-culture-based transformation and *in planta* transformation with antibiotic resistance genes as selectable markers is false-positive transformant shoots or escapees on selection media^14,15^. Consequently, efforts to eliminate false-positive transformants, typically by using GUS assay and PCR analysis of target DNAs, have been deployed. Recently, a novel version of GFPuv, called eYGFPuv which is a mutant of an enhanced yellow GFP-like protein derived from *Chiridius poppei*, was identified and used to generate brilliant green, fluorescent flowers in petunia^16,17^. Positive transgenic shoots were identified under UV light after incorporating an *eYGFPuv* gene construct^17^. However, this approach requires a large 7.5 kb expression cassette, which can create an obstacle during vector construction and cloning, particularly when multiple genes or viral delivery approaches are required. To address this limitation, we reduced the *eYGFPuv* expression cassette by using 1x *eYGFPuv* (2 kb in length) instead of 3x *eYGFPuv* (Fig. 1A) and explored the potential application of 1x *eYGFPuv* in transient expression and stable transgenics in *Arabidopsis*, tobacco, poplar (an important perennial woody bioenergy and landscape plant^18^) and citrus (an important woody fruit crop^19^).

## Results

### Transient expression of eYGFPuv in leaf and root tissues

**Fig. 2:**
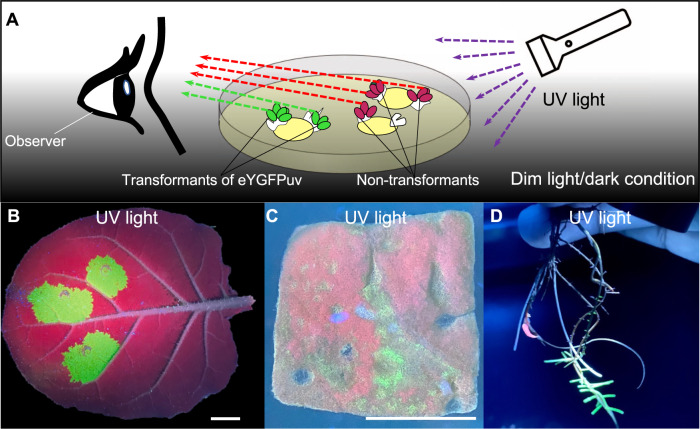
Visualization of eYGFPuv in leaf and root during transient expression. **A** Illustration of visualizing eYGFPuv-expressing samples using UV light. Under long-wavelength UV light (365 nm), eYGFPuv-expressing leaves show green fluorescence, while non-transformed leaves show red autofluorescence. **B** Visualization of eYGFPuv in leaf under UV light, three days post tobacco (*N. benthamiana*) leaf infiltration. Scale bar, 1 cm. **C** Visualization of eYGFPuv in leaf disk of tobacco (*N. tabacum* ‘Xanthi’) under UV light, one week post *Agrobacterium*-mediated in vitro transformation. Scale bar, 1 cm. **D** Visualization of eYGFPuv in the root under UV light, three weeks post poplar root transformation

To achieve strong green florescence during plant transformation, we placed 1x *eYGFPuv* under the control of a cauliflower mosaic virus (CaMV) 35S promoter, a COR47-5’-UTR enhancer^20^ and a HSPT878 terminator^21^ to increase the expression level of eYGFPuv. We then tested the quality of the eYGFPuv plasmid through protoplast transformation in *Arabidopsis* and included a 35S:GFP plasmid as a control. Both GFP and eYGFPuv showed strong green fluorescence under fluorescence microscope, though the fluorescence inensity of eYGFPuv was relatively weaker than that of GFP under same setting (Fig. 1B). As expected, vivid green fluorescence was observed by naked eyes in the Eppendorf tube containing eYGFPuv-expressing protoplasts under UV light, but not in the GFP-expressing protoplasts (Fig. 1B). Next, we tested the activity of eYGFPuv using *Agrobacterium*-mediated leaf infiltration in *Nicotiana benthamiana*. Strong fluorescent signal was observed on the leaf under fluorescence microscope three days post infiltration (Fig. 1C). Next, we examined the infiltrated leaf following the procedure illustrated in Fig. 2A and Supplemental Video 1. As expected, vivid green fluorescence was exhibited on the leaf, along with a red autofluorescence background, under UV light (Fig. 2B). These observations suggest that 1x *eYGFPuv* can generate fluorescence strong enough for detecting eYGFPuv expression during transient expression at both cell and tissue levels. We then examined the expression of eYGFPuv using tobacco (*N. tabacum* ‘Xanthi’) leaf disc transformation. The green fluorescence was observed under the UV light on the leaf discs seven days after infection (Fig. 2C). These results demonstrate that eYGFPuv can be easily visualized both in leaf infiltration and leaf disc transformation.

Because of the wide application of root transformation in producing transformants for assessing gene function, particularly in species recalcitrant to *A. tumefaciens*-mediated transformation^22,23^, we evaluated if 1x *eYGFPuv* could be used for easy and rapid identification of transformed roots. We transformed the 1x *eYGFPuv* expression cassette into the hybrid poplar clone 717 (*Populus tremula* × *P. alba*clone INRA 717-1B4; hereafter ‘717’) using *A. rhizogenes*. We were able to observe green fluorescence signal in roots as early as 14 days post *A. rhizogenes* inoculation. After three weeks, we moved rooted plants to sterile water to allow further root growth and development. Approximately four weeks after inoculation, we were able to distinguish transgenic roots from non-transgenic roots with the aid of the strong eYGFPuv expression (Fig. 2D). Our results suggest that 1x *eYGFPuv* can serve as a reliable marker for selecting transgenic roots without the need of fluorescence microscope and quantifying transformation efficiency in root transformation systems.

### Supplementary Video 1. Visualization of eYGFPuv-expressing events

Vivid green fluorescence was observed in the eYGFPuv-expressing poplar callus excited by a UV light.

### eYGFPuv assisted early selection during tissue culture-based plant transformation

**Fig. 3:**
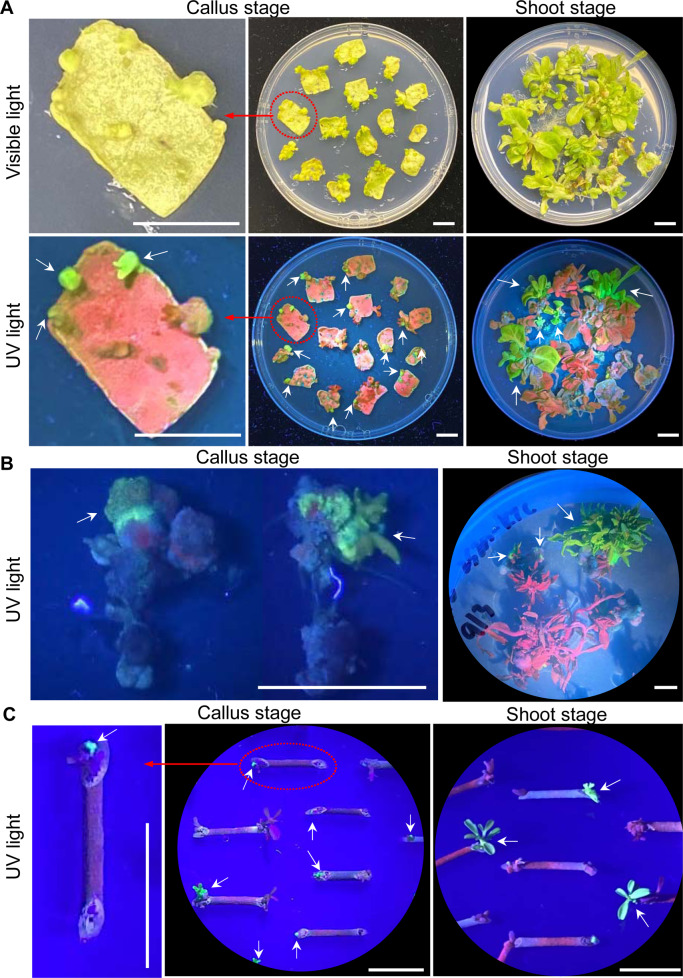
Visualization of eYGFPuv in the early stages of tissue culture-based plant transformation. **A** Visualization of eYGFPuv during callus and shoot stage of tobacco transformation under UV light, two-week and four-week post transformation, respectively. Photos were taken in petri dishes without the lids. **B** Visualization of eYGFPuv during callus and shoot stage of poplar transformation under UV light, four-week and six-week post transformation, respectively. Photos were taken in petri dishes with the lids. **C** Visualization of eYGFPuv during callus and shoot stage of citrus transformation under UV light, five-week and eight-week post transformation, respectively. Photos were taken in petri dishes without the lids. (White arrows indicate eYGFPuv-expressing events; Scale bar, 1 cm)

Tissue culture-dependent plant transformation is an indispensable and widely used method for plant research. To examine 1x *eYGFPuv* expression throughout organogenesis, we initiated *A. tumefaciens*-mediated transformation in herbaceous plant tobacco (*N. tabacum* ‘Xanthi’), woody plants poplar clone 717 and citrus rootstock ‘Carrizo’. In tobacco, calli and small shoots with clear bright green fluorescence were observed two weeks post *Agrobacterium* infection, thus facilitating early selection of transformants (Fig. 3A).

**Fig. 4:**
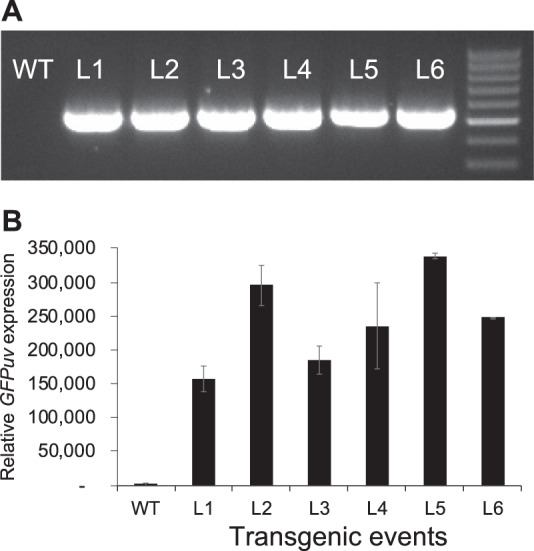
Analysis of eYGFPuv transformants of hybrid poplar clone 717. **A** Genotyping of eYGFPuv transgenic lines. **B** RT-qPCR analysis of eYGFPuv expression level in the transformants and the WT plants. There was no detectable signal in the WT plant sample. The background noise of WT was normalized as 1. Error bars denote standard error over three technical replicates

In comparison with herbaceous plants, the transformation of woody plants is usually more challenging and time consuming. Thus, based on the transgenic tobacco results, we speculate that 1x *eYGFPuv* could play a useful role in early selection of woody plant transformants. Regrettably, we were not able to detect any eYGFPuv signal during the first three-week of callus induction stage in poplar. However, we did observe eYGFPuv-expressing calli and shoots after transferring callus tissue onto shoot induction medium and introducing them to light (Fig. 3B). Hereafter, the eYGFPuv signal maintained at an easily detectable level. Expression of eYGFPuv enabled us to select positive transgenic events under UV light by week five to six post transfection, as opposed to traditional PCR-based genotyping at 10~12 weeks post infection. Ubsequent PCR-based genotyping confirmed the insertion of the *eYGFPuv* gene into the genome in eYGFPuv positive events (Fig. 4A). Interestingly, quantitative reverse transcription PCR (RT-qPCR) revealed a maximum of two-fold difference in the expression level of the *eYGFPuv* gene among six transgenic events that showed similarly eYGFPuv intensity under UV light (Fig. 4B). Citrus transgenic callus and shoots showed similar bright green fluorescence under UV light, while untransformed explants and regenerated tissues showed red autofluorescence (Fig. 3C). Our observations indicate that 1x *eYGFPuv* expression was stable during organogenesis in tobacco, poplar and citrus, and could facilitate the early selection of transgenic callus and shoots.

### Whole-plant level visualization of eYGFPuv in transgenic plants

**Fig. 5:**
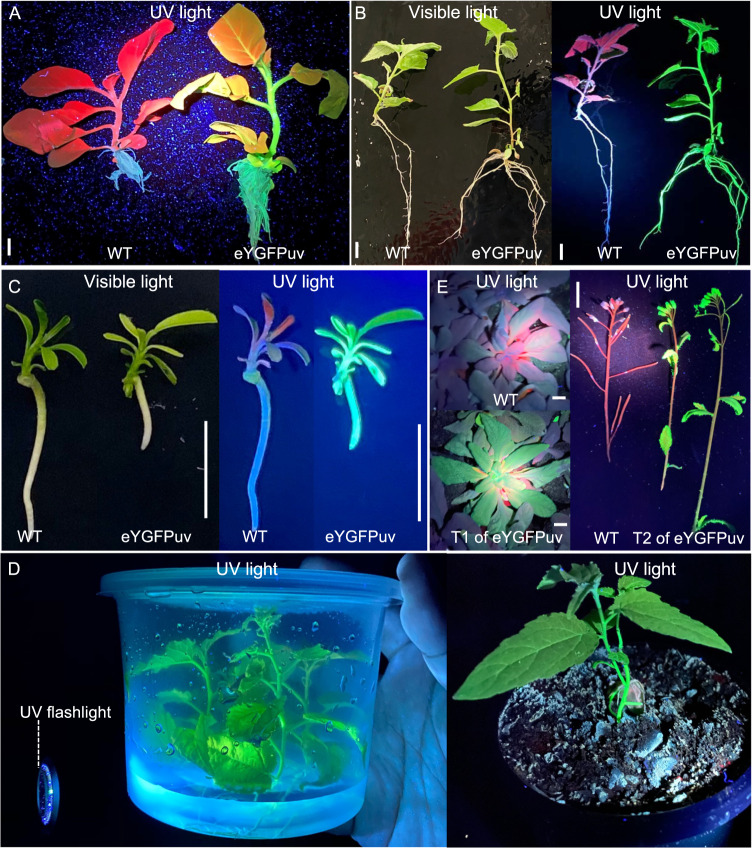
Visualization of eYGFPuv in whole-plant level in different species. **A** Visualization of eYGFPuv in rooted plants of tobacco under UV light, 10 weeks post transformation. **B** Visualization of eYGFPuv in rooted plant of poplar under UV light, three months post transformation. **C** Visualization of eYGFPuv in rooted plant of citrus under UV light, 14 weeks post transformation. **D** Visualization of eYGFPuv in poplar plants placed in medium and soil under UV light, more than three months post transformation. **E** Visualization of eYGFPuv in T1 and T2 of *Arabidopsis* transgenic plants after in-planta transformation under UV light. (Scale bar, 1 cm)

In tobacco, eYGFPuv positive shoots were transferred to rooting medium at six weeks post infection. Compared with the red autofluorescence of the wild-type (WT) tobacco, petioles, stems and roots of the transgenic plants showed a uniform and bright green fluorescence with weaker green signal on the leaf (Fig. 5A). In poplar, eYGFPuv-expressing shoots were transferred to rooting medium ~12 weeks post infection. In contrast to the red autofluorescence in wild type, bright green fluorescence was observed in the whole-plant level of eYGFPuv transformants, including leaves, stems and roots, under UV light (Fig. 5B). Similar to tobacco and poplar, in citrus the whole plant containing 1x *eYGFPuv* appeared vivid green after rooting (Fig. 5C). The easily detectable feature of 1x *eYGFPuv* at the whole-plant level facilitates quick selection of transformed plants grown both in tissue-culture media in the growth chamber and in soil in the greenhouse (Fig. 5D).

**Fig. 6:**
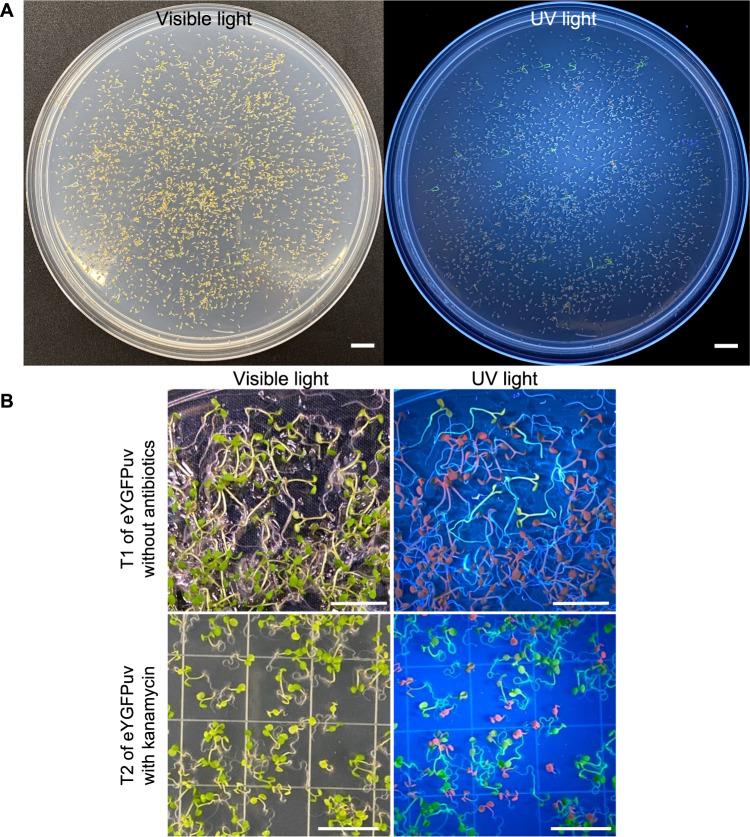
eYGFPuv assisted selection in *Arabidopsis*. **A** Germination of T1 seeds on kanamycin selection plate. **B** Germination of T1 seeds on plant MS plate without antibiotics and germination of T2 seeds on kanamycin selection plate. (Scale bar, 1 cm)

Finally, we studied the performance of 1x *eYGFPuv* in multiple generations of transformed *Arabidopsis*. The T1 plants of transgenic *Arabidopsis* were obtained using floral dip, one of the most commonly used *Agrobacterium*-mediated *in planta* methods. eYGFPuv-expressing T1 seedlings were easily recognized under UV lamp and 87.5% (*n* = 40) kanamycin-resistant seedlings displayed eYGFPuv signals (Fig. 6A). It is highly possible that most of the plants displaying strong eYGFPuv signals contain single-copy transgene because single-copy T-DNA insertion is predominant in *Agrobaterium*-mediated transformation^24,25^. Indeed, one line with strong eYGFPuv signals contains a single-copy transgene, as indicated by a segregation ratio of 1:2.93 (*n* = 212) in T2 generation. Therefore a single copy transgene insertion is adequate for eYGFPuv visualization. This easily detectable feature enables the use of eYGFPuv for antibiotic-free selection of transformants (Fig. 6B). We then monitored eYGFPuv during both vegetative and reproductive growth. In comparison with WT plants, brilliant green fluorescence was observed in rosette leaves, inflorescences, siliques and petals of T1 and T2 transgenic lines without any visible detrimental effects on plant growth and development (Fig. 5E). eYGFPuv positive lines were also confirmed by PCR-based genotyping.

## Discussion

**Fig. 7:**
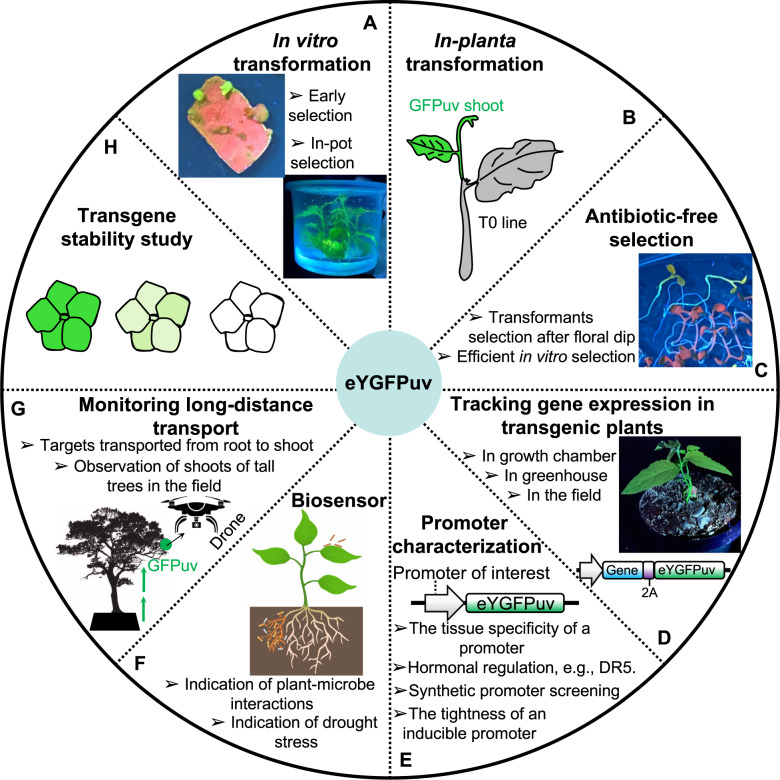
Application of eYGFPuv in plant research. **A** In vitro transformation including early selection and in-pot selection. **B** *In-planta* transformation. eYGFPuv-expressing events can be identified and monitored in real-time. **C** Antibiotic-free selection. It can be directly used for the seed selection post floral dip or a reporter gene in establishing antibiotic-free in vitro transformation. **D** Tracking of gene expression and transgenic plants. It can be used for tracking target gene expression by linking the eYGFPuv to the target gene. It can be used for tracking transgenic lines in different conditions like growth chamber, green house and fields. **E** Promoter characterization including the tissue specificity of a promoter, hormonal regulation, synthetic promoter screening and the tightness of an inducible promoter. **F** Biosensor. It can be used for indicating the invasion of pathogen or the drought stress during plant growth. **G** Monitoring long-distance transport. It can be used for monitoring the transport of targets like proteins from roots to shoots. It can also be used for observing gene expression in the shoot with the help of a drone. **H** Transgene stability study. It can be used for the long-term study of transgene stability

In this study, we demonstrate that green fluorescence can be seen under UV light by naked eyes at both the tissue (e.g., leaf, root) and the whole-plant level through transient gene expression and stable transformation of 1x *eYGFPuv* in herbaceous and woody plants. UV‐excitable eYGFPuv can be easily imaged at a wide range of scales from the sub‐meter level seedlings to whole plants without need for emission filters. Another advantage of eYGFPuv as a reporter is that the eYGFPuv-expressing tissue or whole plants can be directly visualized in a petri-dish or tissue culture vessel without removing the lids (Supplemental Video 1 and Fig. 5D). And eYGFPuv signals can be seen in dim light or dark condition though dark condition (e.g., dark box or dark room) is usually necessary for high-quality imaging. These features suggest that 1x *eYGFPuv* has a wide range of applications in plant science research, as illustrated in Fig. 7.

In general, selectable markers play an important role in plant transformation^13^. In the in vitro transformation, 1x *eYGFPuv* can be used as a selectable marker for in vivo selection of transformants through live imaging without the need for destructive sampling required by PCR-based genotyping or GUS staining, saving time and cost (Fig. 7A). To our knowledge, false-negative transformation error is usually not an issue for plant transformation. It is very common that the reporter, selectable marker or trait genes do not express or express at low level in some transformants due to the well-known “position effect”. These false-negative transformants are usually not selected for further analysis. In contrast, false-positive transformation error caused by antibiotic resistance-based selection is not avoidable. The false-positive transformants can be minimized with the eYGFPuv reporter during plant transformation. Recently, it was reported that transgenic and gene-edited shoots were created through de novo meristem induction without the need for tissue culture and a luciferase reporter was used to indicate the delivery of transgenes^26^. The application of 1x *eYGFPuv* during *in planta* transformation could sidestep the need for substrate and luciferase imaging system and identify the transgenic shoots in real-time (Fig. 7B). Over the past years, the presence of antibiotics in genetically modified plants has raised concerns regarding possible risks for human health and the environment^27^. Here we have shown that 1x *eYGFPuv* can be used for antibiotic-free selection in *Arabidopsis* after flora dip based transformation (Fig. 6B). Similarly, we believe that eYGFPuv can be used for antibiotic-free selection in any other plant species, which can be transformed through floral dip, e.g., *Brassica napus*^28^. It is expected that eYGFPuv can be used as a visual selectable marker for antibiotic-free in vitro transformation (Fig. 7C). Based on the observations in poplar transformation, it is apparent that eYGFPuv-expressing transgenic plants can be easily visualized and tracked in the growth chamber, greenhouse or field (Figs. 5D and 7D), which is particularly useful for the long-term maintenance and evaluation of genetically modified plants. Furthermore, the expression of target genes can be tracked through protein-fusion with an eYGFPuv^29^ (Fig. 7D).

Determination of promoter activity is critical for plant functional genomics and plant synthetic biology research^30,31^. The methods of studying promoter activity are usually based on the expression of a reporter gene driven by the promoter of interest. As a robust reporter, eYGFPuv can be utilized for characterizing promoter properties, such as tissue specificity of a promotor, hormonal regulation of gene expression, synthetic promoter screening and the tightness/leakiness of an inducible promoter (Fig. 7E). In addition, eYGFPuv-based biosensor system can be used for the indication of plant-microbe interactions^6–8^, drought stress and other environmentally triggered reactions (Fig. 7F). We speculate that 1x *eYGFPuv* can serve as a monitor of the long-distance transport of proteins through the plant vascular system^32^. Also, it can be used for monitoring gene expression in the shoots of tall trees in combination with unmanned aerial surveillance (Fig. 7G). Finally, given that the stability of transgene expression is a challenge for plant genetic engineering^33^, eYGFPuv appears suitable for laboratory and field tests over multiple generations for assessing the long-term expression stability of transgenes (Fig. 7H). Furthermore, the eYGFPuv-based biosensor has the potential of enabling advanced plant biotechnology and synthetic biology by combining fluorescent reporters and plant imaging^34^. However, as a new reporter gene, some characteristics of eYGFPuv still remain unclear, e.g., the stability of eGFPuv mRNA and protein in the transgenic plants, such as multiple generations of annual plants or multiple years of perennial plants. The potential of eYGFPuv need to be further exploited in the future.

## Materials and methods

### Construction of vector

To build the 1x *eYGFPuv* expression vector, two gBlocks gene fragments containing enhancer COR47-5’-UTR, eYGFPuv and terminator HSPT878 were synthesized from Integrated DNA Technologies (Coralville, Iowa). The gBlocks were assembled into pGFPGUSPlus (Addgene plasmid # 64401) using the NEBuilder HiFi DNA Assembly Cloning Kit (New England BioLabs, Catalog #E5520S). The presence of eYGFPuv, and its promoter and terminator sequences in the binary vector, was confirmed by Sanger sequencing.

### *Arabidopsis* protoplast transformation

The isolation and transformation of *Arabidopsis* protoplasts was performed as described previously^35^.

### *Arabidopsis* transformation

The *Agrobacterium tumefaciens* strain ‘GV3101’ was used for the transformation of *Arabidopsis* *thaliana* wild type ‘Col-0’ by the floral dip method with modification as described by Yuan et. al^36^.

### Tobacco leaf infiltration

Infiltration of tobacco (*N. benthamiana*) was performed as described by Li^37^.

### Tobacco transformation

The transformation of tobacco (*N. tabacum* ‘Xanthi’) was performed based on a previously described method^38^. In short, after three days of co-cultivation with the *Agrobacterium*, the explants were transferred to the selection medium (MS solid medium with 2 mg/L 6-BA, 200 mg/L Timentin and 100 mg/L kanamycin or 10 mg/L hygromycin). The eYGFPuv positive shoots were transferred to rooting medium (MS basal medium with 200 mg/L Timentin).

### Poplar transformation

The eYGFPuv plasmid was transformed into *A. tumefaciens* strain ‘GV3101’ using electroporation and then transformed into the hybrid poplar clone 717 (*Populus tremula* × *P. alba* clone INRA 717-1B4) following a published method^39^. Throughout the organogenesis stage, kanamycin (50 mg/L) was used to select transgenic events and Timentin (200 mg/L) and cefotaxime (300 mg/L) were used to inhibit the growth of *A. tumefaciens*. eYGFPuv fluorescence was checked regularly using a 365 nm UV flashlight. In addition, we performed genotyping PCR and quantitative reverse transcription PCR (RT-qPCR) to confirm transformants and determine the expression level of eYGFPuv in selected transformants, respectively. After SDS-based genomic DNA extraction^40^, forward primer 5’-CACGGCAACCTCAACG-3’ and reverse primer 5’-CTCGACACGTCTGTGGG-3’ were used for genotyping PCR. After extracting total RNA from leaf samples using Spectrum™ Plant Total RNA Kit (Sigma-Aldrich, MO), we checked RNA quality and quantity with a ND1000 UV–Vis Spectrophotometer (Thermo Fisher Scientific, Waltham, MA). We then treated all RNA samples with DNase (DNase I, amplification grade; Invitrogen, Carlsbad, CA) and used them for cDNA synthesis using SuperScript III Reverse (Invitrogen, Carlsbad, CA), following the manufacturer’s instructions. We performed RT-qPCR with three technical replicates per reaction using Platinum SYBR Green qPCR SuperMix with ROX (Invitrogen, Carlsbad, CA) on a StepOnePlus™ Real-Time PCR System (Applied Biosystems, Foster City, CA). We included two references genes, *EF1-beta* (Potri.009G01860) and *eIF5A* (Potri.018G10730), the primers of which were previously tested in several poplar species^41–43^. The following eYGFPuv-specific primers 5’-GAATCCAATCCACGAGTCCTT-3’ and 5’-ACCTGCTGGTACTCCACTAT-3’ were used. The relative gene expression was determined using the ΔΔCt method.

### Root transformation in poplar

We used the *A. rhizogenes* strain ‘ARqua1’ to produce transgenic roots in the hybrid poplar clone 717 by adapting protocol described by Qiao and Libault^44^. To prepare *A. rhizogenes* culture for root transformation, we transformed the eYGFPuv plasmid into 40 µL competent cells using electroporation at 2.5 kV, 200 Ω and 25 µF and grew the cells in 960 µL liquid LB medium in an incubating shaker at 250 rpm and 28 °C for 1.5 h. We then plated the *A. rhizogenes* culture on solid LB medium containing 50 mg/L kanamycin for two days at 28 °C. After performing colony PCR confirmation of the presence of the eYGFPuv plasmid, we grew an individual colony in 50 ml LB medium containing 50 mg/L kanamycin overnight. The *A. rhizogenes* cells were then pelleted by centrifuging at 4000 rpm for 10 min and then re-suspended in freshly prepared dilution buffer containing 3.92 mg/L acetosyringone and 1 mL/L DMSO at an OD600 nm of 0.3–0.6. To generate roots, we collected four-week-old shoot tips sprouting from trimmed greenhouse poplar plants and then wounded the bottom 1.5 cm region of the stems with multiple fine cuts. The wounded shoot tips were then inserted into autoclaved rockwool blocks, each of which was pre-soaked with 40 ml diluted *A. rhizogenes* culture. The inoculated shoot tips were grown under the condition of 22 °C, 70% humidity and under 16 h light/8 h dark photoperiod and watered with 40 ml sterile H_2_O every five to six days. About two to three weeks after inoculation, rooted shoot tips were carefully removed from individual rockwool blocks and kept in sterile H_2_O for future analysis.

### Citrus transformation

The transformation of ‘Carrizo’ citrange rootstock (*Citrus sinensis* ‘Washington’ sweet orange × *Poncirus trifoliata*) was performed as previously described^45^ with some modifications. In brief, the fresh *A. tumefaciens* strain ‘GV3101’ or ‘EHA105’ solution was resuspended in a liquid MS medium containing 30 g/L sucrose and 50 mg/L acetosyringone. We cut the four-week-old, etiolated epicotyls into 1 cm length segments and soaked them in resuspension solution for 20 min. The explants were blotted dry on sterile filter paper and then transferred onto solid MS medium containing 50 mg/L acetosyringone. After three days of co-cultivation, we transferred the explants to MS medium containing 30 g/L sucrose, 3.0 mg/L 6-BA, 100 mg/L Timentin and 75 mg/L kanamycin and incubated them under a light intensity of 60 μmol/m^2^ s^−1^ with a 16 h light/8 h dark photoperiod. All the explants were inspected for GFP fluorescence under the ong-wavelength (365 nm) UV light. The GFP-positive shoots were transferred to a rooting medium consisting of 1/2 MS medium with 30 g/L sucrose, 0.1 mg/L NAA and 0.1 mg/L IBA, Timentin 100 mg/L and activated carbon 0.6 g/L.

### UV flashlight

Different UV flashlight sources were tested in this study: LIGHTFE UV302D (365 nm) and uvBeast UVB-V3-365 (365 nm), which are suitable for small and big samples, respectively.

## Supplementary information

Supplemental Video 1

## Data Availability

The plasmids will be available at Addgene.
